# Concerning the Symptomatology of Thoracic Aneurysms

**Published:** 1903-10

**Authors:** Truman A. Parker

**Affiliations:** Richmond, Va., Instructor in Physiology, University College of Medicine


### CONCERNING THE SYMPTOMATOLOGY OF THORACIC ANEURYSMS.*

By TRUMAN A. PARKER, M.D., RICHMOND, VA.,
Instructor in Physiology, University College of Medicine.

We all know that an aneurysmal tumor of the ascending arch will present a train of symptoms differing somewhat from those consequent upon a tumor in the course of the descending aorta. But I shall leave to the professional diagnosticians the localization symptomatology of this condition, and speak of the symptoms arising from an aneurysm of the aorta in the thorax, in a general way and to the general practitioner. His overcrowded mind does well to maintain a clear conception of the varied and numerous lesions which may be caused by an aneurysmal tumor of the thoracic aorta. He knows that treatment is practically the same.

Personal observation, apart from the experience of others, has forced upon me the conclusion that many a patient has received anti-rheumatic, anti-neuralgic and anti-bronchitic treatment *ad libitum*, because he was innocent enough to carry a thoracic aneurysm engendering pressure symptoms. It is my earnest hope this evening, to obtain mental entertainment on the part of some of my hearers, for the idea of thoracic aneurysm in any condition where intrathoracic pressure symptoms are manifest, or the physical signs enumerated below present themselves.

You will doubtless be impressed by the undercurrent of doubt and uncertainty which permeates this reading. It must needs be so. The symptom complex of this condition is as varied in degree

*Read before the Richmond Academy of Medicine and Surgery, July 28, 1903.

as it is in character. It is in recognition of this fact that Bramwell, in classifying aortic aneurysms, gives first place to "those which are entirely latent, give no physical signs"; and again, "secondly, those giving signs of intrathoracic pressure, but in which the nature of the cause can not be ascertained"; and thirdly, "those which form distinct tumors and give well-marked pressure symptoms and external signs." Let us not then postpone the consideration of aneurysm until there appears a pulsating distensile tumor in the neighborhood of the upper portion of the sternum associated with intense, boring, aching pain. Such manifestations are the concomitants of thoracic aneurysm in a limited number of cases only.

The tumor may project posteriorly; it may ulcerate into the esophagus, a portion of the respiratory tract, the pericardium and even into one of the great veins of this region. Bloody expectorations then, may be due to nothing more nor less than an aortic aneurysm. Musser speaks of a case coming under his observation which had been treated for pulmonary tuberculosis. This treatment was based upon the following symptoms: emaciation, fever and loose cough with bloody expectorations. Autopsy revealed aneurysmal tumor rupturing into the bronchus, which it had previously occluded causing bronchiectasis, etc.

Nor is pain, which it is well to remember as a very constant symptom, always of the same character. At times it is sharp and lancinating, occurring in paroxysms. Again, it may be anginal in character, or neuralgic, extending down the arm into the neck, or along the course of the intercostal nerves,—symptoms of nerve pressure from the growing tumor. Tracheal tugging, or Oliver's sign, is another well-remembered symptom of this condition; but in remembering this sign, which is only present when the tumor presses upon the left bronchus as it passes under the arch, or when it is adherent to the trachea, we are prone to forget dyspnea and dysphagia from pressure upon the trachea and esophagus.

So, too, we are famaliar with the "brassy cough," paroxysmal and ringing in character, laryngeal in origin, and dry, due to pressure upon the recurrent laryngeal nerve. But we ignore the irritative, hacking cough with thin, watery expectoration resulting from pressure upon the trachea proper; or the loose cough with copious, ropy discharge, when a bronchus has been compressed and bronchiectasis, with fever, has set in. Pressure upon the recurrent laryngeal may also produce dyspnea, with stridor, aphonia, hoarseness, and "a peculiar monotone and inability to reach a high note," due to paralysis of one or both vocal cords. Not that this entire train of symptoms is always present when the laryngeal nerve is compressed. There is often no other symptom save the brassy cough. There may be some other *one* symptom, *e. g.*, Moritz Schmidt has reported a series of fifty-four cases, in all of which the patients first consulted him on account of *hoarseness*. Thirty-eight showed paralysis of the left cord, the one most frequently involved. In one case both were involved.

Occasionally the tumor will encroach upon the thoracic duct, causing inanition and wasting, which may divert one's attention to phthisis. Again, it may partly occlude one of the venæ cavæ, with resulting engorgement of the head, neck, thorax, etc., perhaps with edema. Occasionally there is seen clubbing of the fingers of one hand with incurvation of the nails and congestion, due to venous engorgement.

The sympathetic system has a minor rôle in this symptomatology. Pressure-irritation gives dilatation of the pupil on one side, with pallor; while pressure-paralysis results in pupillary contraction and hyperemia. Unilateral sweating is sometimes present.

The physical signs are most varying in degree. We have noticed that they may be altogether lacking. On the other hand, they may be most pronounced. Not infrequently inspection reveals a pulsation. It may be slight and without visible swelling, or marked, diffuse and heaving in character. When present, it is synchronous with cardiac systole. There may be a pronounced tumor with ulcerating skin and slight leakage. These signs are chiefly found above the third right rib and to the right of the sternum, sometimes to the left, some over that bone. Pulsation is quite often noted in the suprasternal notch, or above the clavicle when the innominate is involved. The apex beat is displaced downward and to the left. It is frequently heaving in character.

Palpation should be performed thoroughly, single handed and bimanually, on full inspiration and full expiration. Usually, the tumor, when appearing externally, is hard and non-expansile from fibrinous deposit. It may be soft and compressible. Thrill, systolic in time, may be present; also a diastolic shock, which, when present, is diagnostic.

*Percussion* furnishes very reliable evidence when the tumor is not too deeply seated or small in size. Accordingly, dullness is obtained in varying degree. When the tumor is superficial, it may yield flatness; or relative dullness only may be obtained. It is usually found on either side of the sternum and over the same; sometimes in the left interscapular region. Percussion both in the upright and in the recumbent posture should be performed. The occurrence of severe paroxysms of coughing or the complaint of pain while percussing the anterior chest Eichhorst considers almost diagnostic. The difference between the percussion note and the shape of the dullness on full inspiration and full expiration may influence a wavering judgment.

*Auscultatory percussion* is regarded of much value. Aneurysmal tumor may be present without thrill or murmur, but yields signs of dullness on percussion.

*Auscultation.* Murmur, when present, is usually best heard over the tumor or abnormal pulsation. It is usually systolic in time and transmitted in the direction of the vessels. It may be best heard over the vessels in the neck or in the course of the aorta. Sometimes the double murmur of associated aortic regurgitation is present. Occasionally, only a diastolic murmur is heard. As a rule the aortic second sound is markedly accentuated and ringing in character.

I have intentionally omitted fluoroscopy up to this point, in order to emphasize its importance. By its means a condition of deeply seated tumor may be diagnosed when paucity of other symptoms confound us.

*Diagnosis.* Of this I shall speak but scantily. It must be based upon the etiological factors and the pathological condition of the vessels; upon the occurrence of pain, of pressure-symptoms and of the physical signs already alluded to. Among the chief conditions confusing the diagnosis are the following: Carcinomatous disease of the mediastinum. This is to be differentiated by history and glandular involvement elsewhere on the one hand, and expansile pulsation on the other. Some of the physical signs, too, are of assistance. Benign tumors are rare in this locality. Pulsation from simple aortic regurgitation with dilatation must be considered; also neurotic pulsation in anemic individuals.

In pulsating empyema percussion-dullness is at the base of the chest and quite extensive—usually upon the left side and at a distance from the median line. Pulmonary phthisis is to be differentiated by sputum examination and a consideration of the vascular and other signs of aneurysm. Before closing, I would call attention to Musser's opinion, that inasmuch as aneurysm may be present without diagnostic physical signs, while, on the other hand, pressure-symptoms may be in abeyance, if one of the two is present in the male subject past forty, with a previous history of gout, syphilis, alcoholism or muscular strain, the probability is that aneurysm exists.

*Literature.* Musser's Med. Diagnosis, Sajous' Analyt. Cyc. of Pract. Med., Butler's Diag. of Inter. Med., Bishop on Ear, Nose and Throat, Hare's Med. Diag., etc.

